# Variability in the Composition of Pacific Oyster Microbiomes Across Oyster Families Exhibiting Different Levels of Susceptibility to OsHV-1 μvar Disease

**DOI:** 10.3389/fmicb.2019.00473

**Published:** 2019-03-11

**Authors:** William L. King, Nachshon Siboni, Nathan L. R. Williams, Tim Kahlke, Khue Viet Nguyen, Cheryl Jenkins, Michael Dove, Wayne O’Connor, Justin R. Seymour, Maurizio Labbate

**Affiliations:** ^1^The School of Life Sciences, University of Technology Sydney, Ultimo, NSW, Australia; ^2^Climate Change Cluster, University of Technology Sydney, Ultimo, NSW, Australia; ^3^NSW Department of Primary Industries, Elizabeth Macarthur Agricultural Institute, Menangle, NSW, Australia; ^4^NSW Department of Primary Industries, Port Stephens Fisheries Institute, Port Stephens, NSW, Australia

**Keywords:** *Crassostrea gigas*, microbiome, ostreid herpesvirus, disease resistance, *Vibrio*, POMS

## Abstract

Oyster diseases are a major impediment to the profitability and growth of the oyster aquaculture industry. In recent years, geographically widespread outbreaks of disease caused by ostreid herpesvirus-1 microvariant (OsHV-1 μvar) have led to mass mortalities among *Crassostrea gigas*, the Pacific Oyster. Attempts to minimize the impact of this disease have been largely focused on breeding programs, and although these have shown some success in producing oyster families with reduced mortality, the mechanism(s) behind this protection is poorly understood. One possible factor is modification of the *C. gigas* microbiome. To explore how breeding for resistance to OsHV-1 μvar affects the oyster microbiome, we used 16S rRNA amplicon sequencing to characterize the bacterial communities associated with 35 *C. gigas* families, incorporating oysters with different levels of susceptibility to OsHV-1 μvar disease. The microbiomes of disease-susceptible families were significantly different to the microbiomes of disease-resistant families. OTUs assigned to the *Photobacterium*, *Vibrio*, *Aliivibrio*, *Streptococcus*, and *Roseovarius* genera were associated with low disease resistance. In partial support of this finding, qPCR identified a statistically significant increase of *Vibrio*-specific 16S rRNA gene copies in the low disease resistance families, possibly indicative of a reduced host immune response to these pathogens. In addition to these results, examination of the core microbiome revealed that each family possessed a small core community, with OTUs assigned to the *Winogradskyella* genus and the *Bradyrhizobiaceae* family consistent members across most disease-resistant families. This study examines patterns in the microbiome of oyster families exhibiting differing levels of OsHV-1 μvar disease resistance and reveals some key bacterial taxa that may provide a protective or detrimental role in OsHV-1 μvar disease outbreaks.

## Introduction

The Pacific oyster, *Crassostrea gigas* is a globally cultivated oyster species, but the cultivation of this species has been increasingly impacted by disease events (Azéma et al., 2015). These disease events are largely caused by viral and bacterial etiological agents (Friedman et al., 2005; Garnier et al., 2007; Segarra et al., 2010; King et al., 2019), but in some instances no clear etiological agent is identifiable (Go et al., 2017; King et al., 2018). A major pathogen of *C. gigas* is the ostreid herpesvirus 1 (OsHV-1), and its micro variant form (OsHV-1 μvar) (Davison et al., 2005; Segarra et al., 2010). This virus has caused severe mortality outbreaks over the last two decades (Friedman et al., 2005; Burge et al., 2006; Segarra et al., 2010; Jenkins et al., 2013; Mortensen et al., 2016), with some outbreaks resulting in over 90% mortality and leading to the death of many millions of oysters (ASI, 2015).

To combat the impact of OsHV-1 μvar, a variety of approaches including modifying husbandry practices (e.g., increased oyster cultivation height) and breeding disease resistant oysters have been applied, with varying degrees of success (Dégremont, 2011; Paul-Pont et al., 2013; Whittington et al., 2015). Breeding programs generally involve breeding oyster genetic lines that have greater survival rates following exposure to OsHV-1 (Dégremont, 2011, 2013; Dégremont et al., 2015b; Camara et al., 2017). While these breeding programs have shown some success, resistant families still experience varying degrees of mortality (juvenile oysters 5–19%; larvae up to 86%) (Dégremont, 2011; Dégremont et al., 2013, 2016b), and the mechanism(s) underpinning resistance are often not easily distinguishable.

A number of studies have characterized the physiological and immunological factors driving OsHV-1 and OsHV-1 μvar resistance in selectively bred oysters (Sauvage et al., 2009; Dégremont, 2011, 2013; Azéma et al., 2015; Dégremont et al., 2015a, 2016a,b). Factors such as increased oyster size and weight are associated with increased resistance to infection, but why this occurs is currently unclear (Dégremont, 2013; Dégremont et al., 2015b). Other studies have determined that resistant oysters have a greater capacity to clear OsHV-1 from their tissues and supress virus replication (Dégremont, 2011; Segarra et al., 2014). When examined from an immunologic perspective, resistant oysters appear to have greater capacity to induce autophagy genes when infected by OsHV-1 compared to susceptible oysters (Moreau et al., 2015).

Another contributing factor that has received less attention is the oyster microbiome. A previous study has shown that despite being positive for OsHV-1 μvar, antibiotic-treated oysters displayed significantly reduced mortalities in comparison to untreated oysters (Petton et al., 2015). Furthermore, the total bacterial load, including the *Vibrio* community, is significantly elevated following OsHV-1 μvar infection and this elevation is necessary to cause mortality (de Lorgeril et al., 2018). *Vibrio* bacteria are commonly isolated from OsHV-1 infected oysters (Segarra et al., 2010; Garcia et al., 2011; Jenkins et al., 2013; Keeling et al., 2014), with a recent study concluding that OsHV-1 μvar infection causes immune-suppression of the oyster host, allowing opportunistic bacteria (including *Vibrio* species) to infect the oyster (de Lorgeril et al., 2018). In other organisms, studies have implicated the host microbiome as modulating the immune system, suggesting it is critical in host defense and overall health (reviewed by Shreiner et al., 2015) and in influencing host behavior (Shin et al., 2011).

To better understand how breeding for OsHV-1 μvar disease resistance affects the *C. gigas* microbiome and to elucidate whether specific taxa are associated with susceptibility and resistance, we examined the microbiome of 35 *C. gigas* families with varying degrees of disease-resistance. To remove the confounding effects of time and location, these oysters were deployed at a single location and sampled at the same time. In addition, the comparison of distinct *C. gigas* families provided the opportunity to determine whether they harbored distinct microbial community assemblages and whether persistent bacterial taxa (core microbiome) common across the families could be identified.

## Materials and Methods

### Sources and Sampling of *C. gigas*

Australian Seafood Industries (ASI) is an oyster aquaculture industry-owned company that since the first OsHV-1 μvar outbreak in 2010 has been breeding *C. gigas* families for OsHV-1 μvar disease resistance through field exposure. In 2016, ASI deployed 35 (*n* = 35) 5^th^ generation families (5 consecutive years of biparental breeding) of juvenile *C. gigas* into three areas known to harbor the OsHV-1 virus, the Georges River (NSW, Australia; 34.035S, 151.145E), Pipe Clay Lagoon (TAS, Australia; 42.970S, 147.525E) and Pittwater (TAS, Australia; 42.802S, 147.509E) (Kube et al., 2018). Based on these field disease-exposure studies, expected breeding values (EBVs) were calculated by ASI. These EBVs are an estimation of how well the oysters will perform for a particular trait and the likelihood of passing those traits to their progeny. For the purposes of this study, families were classified into ‘resistance groups’ (RG) based on their OsHV-1 μvar disease resistance EBV. Families with an EBV greater than 0.6 were placed into RG1 (high disease-resistance), those with an EBV greater than 0.3 and less than 0.6 were placed into RG2 (medium disease-resistance), and families with an EBV less than 0.3 were placed into RG3 (low disease-resistance) (Table 1). The estimated heritability is the likelihood of the offspring demonstrating a particular trait, in this case OsHV-1 μvar disease resistance. Resistance is determined by the combination of many genes, since the stock used are derived from a number of genetically distinct families, each family differs in its resistance, and crosses between families differ.

**Table 1:** Expected breeding value ranks for the studied oyster families including OsHV-1 μvar disease-resistance.

	OsHV-1	
Family	μvar	Resistance	Meat	Depth	Shell	Oyster	Width
line	resistance	group (RG)	condition	index	length	weight	index
F_01	8	RG2	22	6	28	7	7
F_02	25	RG3	20	1	29	10	10
F_03	6	RG2	17	18	21	13	13
F_07	16	RG2	6	4	34	1	1
F_10	26	RG3	16	10	32	5	5
F_11	24	RG3	11	7	31	6	6
F_15	29	RG3	6	4	34	1	1
F_16	31	RG3	3	14	11	23	23
F_19	17	RG2	17	18	21	13	13
F_20	28	RG3	4	20	9	13	13
F_23	15	RG2	13	28	19	19	19
F_25	20	RG2	6	21	19	20	20
F_26	32	RG3	12	8	25	22	22
F_27	22	RG3	25	2	27	17	17
F_29	7	RG2	27	31	2	35	35
F_30	18	RG2	23	30	3	33	33
F_35	34	RG3	1	27	10	8	8
F_36	10	RG2	9	24	17	24	24
F_37	12	RG2	4	9	30	11	11
F_39	27	RG3	2	15	17	9	9
F_40	11	RG2	17	12	26	21	21
F_43	19	RG2	20	10	21	28	28
F_51	23	RG3	14	28	13	12	12
F_61	30	RG3	14	35	1	34	34
F_62	33	RG3	24	24	7	17	17
F_65	13	RG2	30	31	3	30	30
F_66	1	RG1	30	31	3	30	30
F_67	9	RG2	30	31	3	30	30
F_68	3	RG1	30	16	15	25	25
F_69	5	RG1	30	16	15	25	25
F_72	2	RG1	35	21	13	16	16
F_77	4	RG1	26	24	8	25	25
F_80	21	RG3	29	23	12	29	29
F_84	14	RG2	28	2	33	3	3
F_86	35	RG3	10	13	24	4	4


*Survival data was found after deployment in three different OsHV-1 μvar positive estuaries across Australia. Expected breeding values (EBVs), including OsHV-1 μvar disease-resistance, are shown as a rank number out of 35*.

In addition to disease-resistance, EBVs of other oyster traits were also provided by ASI. These traits include: meat condition, the ratio of wet meat to the total weight; depth index, the ratio of shell depth to shell length; shell length; oyster weight, including the oyster shell; and width index, the ratio of shell width to shell length. As EBV’s are proprietary information, rather than providing absolute values for each index, we generated a ‘rank’ system to categorize families according to each index, with ranks of 1 being the highest (Table 1).

For this microbiome study, the families were deployed into the Georges River (34.035S, 151.145E) on the 16^th^ of August 2016 and sampled 2 months after deployment date. The 2-month deployment time was the first opportunity to sample the deployed juvenile oysters and was sufficient time to ensure no evidence of disease or morbidity. Oysters were deployed in a resolvable incomplete block design to account for micro-geographic variation, blocks were subsections of a replicate and there were three replicates for each family, with each family stocked into a subsection of the tray (Kube et al., 2018). Five oysters from each of the 35 families (total = 175 samples) were sampled and immediately placed on ice and transported to the laboratory where they were stored at -80°C until further processing.

### DNA Extraction, Sequencing, and Bioinformatics

The outer shell of the five sampled oysters was rinsed under running tap water to remove any remaining mud and debris. Defrosted oysters were then shucked with sterilized shucking knifes and approximately 25 mg of adductor muscle tissue was aseptically removed using sterile scalpel blades.

The Qiagen DNeasy blood and tissue kit (catalogue: 69506) was used to extract DNA samples, as per the manufacturer’s instructions. Microbial community composition within samples was subsequently assessed using 16S rRNA amplicon sequencing, whereby the ribosomal 16S rRNA V1–V3 region was targeted using the 27F (5′-AGAGTTTGATCMTGGCTCAG-3′) and 519R (5′-GWATTACCGCGGCKGCTG-3′) primer pair. The PCR cycling conditions were as follows: 95°C for 3 min, 25 cycles of 95°C for 30 s, 55°C for 30 s and 72°C for 30 s, and a final extension at 72°C for 5 min. Amplicons were sequenced using the Illumina MiSeq platform (2 × 300 bp) using standard approaches (Ramaciotti Centre for Genomics at the University of New South Wales, Sydney, NSW, Australia). Raw data files in FASTQ format were deposited in the NCBI Sequence Read Archive (SRA) under the Bioproject number PRJNA497763.

Briefly, 16S rRNA paired-end DNA sequences were joined using Flash (Magoc and Salzberg, 2011) and subsequently trimmed using Mothur (Schloss et al., 2009) (Parameters: maxhomop = 5, maxambig = 0, minlength = 432, maxlength = 506). The resulting fragments were clustered into operational taxonomic units (OTUs) at 97% sequence identity, and chimeric sequences were identified using vsearch (Rognes et al., 2016). Taxonomy was assigned in QIIME (Caporaso et al., 2010) using the uclust algorithm (Edgar, 2010) against the Silva v128 database. Mitochondrial and chloroplast data were filtered out of the dataset and the remaining data were rarefied to allow for even coverage across all samples (Supplementary Data Sheet 2). OTUs representing less than 0.1% relative abundance in an individual sample were also filtered from the dataset (Supplementary Tables 1, 2).

### Core Microbiome Analysis

To determine whether a core oyster microbiome could be characterized, we examined the microbiome of oysters at three different thresholds. First, for individual families, then for RGs, then for all samples together. A core OTU was defined as an OTU that was present in at least all but one replicate (to account for outliers) within a family. To achieve this, the panbiom.py script was used as detailed in Kahlke (2017). Briefly, the final biom file generated during the QIIME analysis was used in conjunction with a treatment file that identifies which samples are replicates within a family. The panbiom.py arguments were as follows: a replicate threshold of 1 (-r parameter) and an outlier threshold of ‘x’ (-x parameter). The -x parameter treats the replicate threshold value as an outlier threshold value, simply put, it can be absent in one replicate sample (indicated by -r = 1 and -x = x).

### Quantitative PCR (qPCR)

Due to the potential role of *Vibrio* in OsHV-1 μvar disease dynamics (Segarra et al., 2010; Jenkins et al., 2013; Lemire et al., 2015; Petton et al., 2015; de Lorgeril et al., 2018), quantitative PCR (qPCR) was used to examine patterns in *Vibrio* abundance across the RGs. qPCR was performed using an epMotion 5075l Automated Liquid Handling System on a Bio-Rad CFX384 Touch Real-Time PCR Detection System with a six-point calibration curve and negative controls on every plate. The calibration curve was built from a known amount of amplicon DNA measured by Qubit, followed by a 10-fold dilution to fill out the calibration curve. All sample analyses were performed with three technical replicates, using the following reaction mixture: 2.5 μL iTaq Universal SYBR Green supermix, 0.4 μM of each forward and reverse primer, 1 μL of diluted (1:15) template DNA, and the remainder made up with water. To quantify abundance of the *Vibrio* community, the *Vibrio*-specific 16S rRNA primers Vib1-f (5′-GGCGTAAAGCGCATGCAGGT-3′) and Vib2-r (5′-GAAATTCTACCCCCCTCTACAG-3′) were used (Thompson et al., 2004; Vezzulli et al., 2011; Siboni et al., 2016). The qPCR cycling conditions were as follows: 95°C for 3 min followed by 45 cycles of 95°C for 15 s and 60°C for 1 min. The resulting data were normalized to both elution volume (200 μL) and tissue weight. A coefficient of variation (CV) was then calculated for the technical triplicates, and samples with CV > 10% were removed from the analysis. A melting curve was added to the end of every run to confirm the presence of a single PCR product.

### Statistical Analysis

Comparisons of alpha diversity were performed with a one-way ANOVA followed by a Tukey’s pairwise test. Normalized [square root (x)] data were used to compare community compositions using a non-metric multidimensional scaling analysis (nMDS) with a Bray–Curtis similarity index. To determine significantly different microbial assemblage between families and RGs, and to compare qPCR data, a one-way PERMANOVA was used. To examine which OTUs contributed to differences between RGs, a SIMPER analysis with a Bray–Curtis similarity index was used. To define associations between breeding values and OTUs, breeding values were normalized (x-mean/standard deviation) and used within a canonical correspondence analysis (CCA). All analyses were performed using the PAST statistical software (Hammer et al., 2001). To determine whether an OTU was significantly elevated in a particular RG, the group_significance.py script using the default analysis (Kruskal–Wallis ANOVA) was used in QIIME. To examine correlations between EBVs, we performed a maximal information-based non-parametric exploration (MINE) analysis (Reshef et al., 2011).

## Results

### The *C. gigas* Microbiome

**FIGURE 1:**
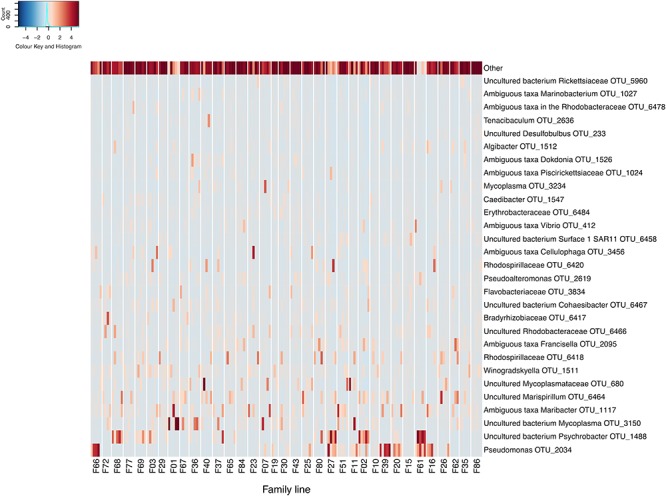
Heatmap of scaled OTU relative abundance for the 30 most abundant OTUs, as well as the remaining summed lowly abundant OTUs. Families ordered by OsHV-1 μvar disease-resistance. Heatmap was made using the R statistical environment using scaled data with the gplots and RColorBrewer packages (Neuwirth, 2014; Warnes et al., 2016; R Core Team, 2017).

Following data filtering and rarefication, a total of 3294 OTUs were observed across the entire dataset, and of these, 68.5% occurred at below 1% of the total relative abundance. Conversely, across all samples and spanning all RGs, a member of the *Pseudomonas* genus (OTU 2034) was found to be the most relatively abundant OTU comprising 5.6% of the bacterial community. This was followed by OTUs matching an uncultured bacterium in the *Psychrobacter* genus (OTU 1488) and an uncultured bacterium in the *Mycoplasma* genus (OTU 3150), which represented 4.8 and 4% of the *C. gigas* microbiome across the whole dataset respectively (Figure 1).

### Variability in the *C. gigas* Microbiome Across Different Resistance Lines

To determine whether breeding for disease resistance influences the *C. gigas* microbiome, the microbiome of oysters assigned to RGs were characterized and compared. Alpha diversity, quantified using Shannon’s diversity index was significantly higher in RG2 when compared to the RG3 RG (*F*_(1,141)_ = 6.8, *p* = 0.025), but did not vary significantly when compared to RG1 (*F*_(1,93)_ = 0.4, *p* = 0.51). Species richness (Chao1) did not differ significantly between any of the RGs (RG1 vs. RG2 – *F*_(1,93)_ = 0.03, *p* = 0.85; RG1 vs. RG3 – *F*_(1,94)_ = 1.3, *p* = 0.26; RG2 vs. RG3 – *F*_(1,141)_ = 1.08, *p* = 0.30).

Comparisons of microbiome composition (beta diversity) across different RGs revealed that the microbiomes of RG1 and RG2 were both significantly different to the least disease resistant group, RG3 (*p* = 0.019 and *p* = 0.0001; *F*_(1,94)_ = 1.47 and *F*_(1,141)_ = 2.93 respectively). No significant difference was found between the microbiomes of RG1 and RG2 (*F*_(1,93)_ = 1.29, *p* = 0.055). Statistical comparisons between RG2 and RG3 appeared to be stronger than those between RG1 and RG3, possibly due to more families being assigned to RG2, therefore potentially adding more microbiome variability to this group. No clear dissimilarity in the microbiome of the RGs was apparent in a 3D nMDS (Stress = 0.34), or a PCoA (Supplementary Figure 1). SIMPER comparisons showed that the composition of the microbiomes associated with RG1 and RG2 were 81.83 and 82.12% dissimilar to RG3 respectively (Supplementary Tables 3, 4).

**FIGURE 2:**
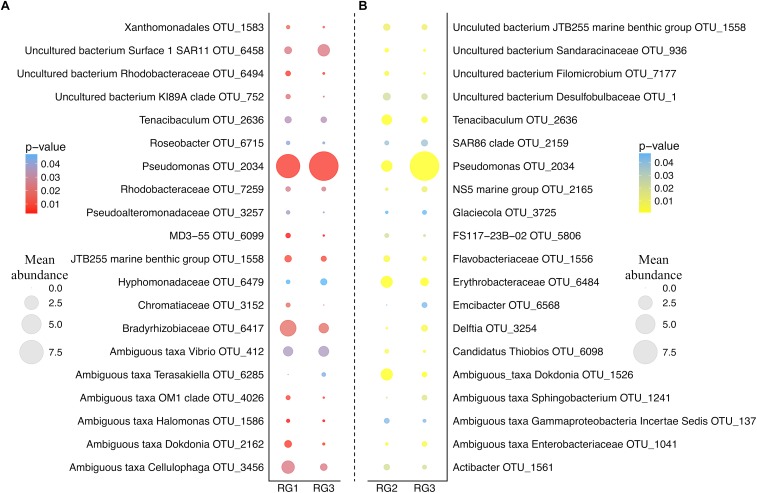
Bubble plot of group_significance.py analysis results using the default Kruskal–Wallis parameters. **(A)** Represents the comparison between RG1 and RG3. **(B)** Represents the comparison between RG2 and RG3. Color represents the strength of the *p*-value. Size represents the mean relative abundance of that OTU across the whole resistance group (RG). OTUs assigned to the genus and species level were chosen, and those 20 most abundant from each RG are displayed.

As the RG with the lowest level of disease-resistance (RG3) was found to have a significantly different microbial assemblage to both RG2 and RG1, we examined which OTUs were responsible for driving the differences in microbiome structure between these groups (Figure 2). An OTU assigned to the *Pseudomonas* genus (OTU 2034; the most abundant OTU in the entire dataset) was over-represented in the RG3 microbiome relative to both RG1 (*H*_(1,94)_ = 7.6, *p* = 0.0058) and RG2 (*H*_(1,141)_ = 15, *p* = 0.00011). Conversely, an OTU assigned to the *Tenacibaculum* genus (OTU 2636) and two separate OTUs assigned to the *Dokdonia* genus (OTUs 2162 and 1526) were all significantly under-represented in RG3 (*Tenacibaculum* RG1 *H*_(1,94)_ = 4.5, *p* = 0.033 and RG2 *H*_(1,141)_ = 15.2, *p* = 0.0056; *Dokdonia* RG1 *H*_(1,94)_ = 7.7, *p* = 0.0001 and RG2 *H*_(1,141)_ = 30.3, *p* < 0.0001).

Notably, a member of the *Vibrio* genus (OTU 412) was found to be significantly over-represented in the least disease-resistant group (RG3) relative to the most disease-resistant group (RG1) (*H*_(1,94)_ = 4.4, *p* = 0.036). Due to the previously demonstrated importance of *Vibrio* species in OsHV-1 μvar infection (Segarra et al., 2010; Jenkins et al., 2013; de Lorgeril et al., 2018), we subsequently employed a *Vibrio*-specific 16S rRNA qPCR assay to compare total abundances of *Vibrio* across RGs. A significant elevation of *Vibrio* 16S rRNA gene copies was observed in RG3 compared to RG1 (*F*_(1,94)_ = 2.86, *p* = 0.027) and RG2 (*F*_(1,141)_ = 3.25, *p* = 0.014) (average of 179, 107, and 75 gene copies mg of tissue^-1^ respectively; Supplementary Figure 2). Furthermore, OTUs assigned to the *Vibrio* genus were significantly elevated in RG3 when compared to RG1 (*F*_(1,94)_ = 4.27, *p* = 0.011), but not RG2 (*F*_(1,141)_ = 2.48, *p* = 0.07). To determine the extent of whether *Vibrio* OTUs were driving the differences between RG1 and RG3 microbiomes, OTUs assigned to the *Vibrio* genus were removed and the RG beta diversity comparison was reperformed. When doing this, we observed a slight weakening of the statistical comparison between RG1 and RG3, from (*F*_(1,94)_ = 1.47, *p* = 0.019) to (*F*_(1,94)_ = 1.46, *p* = 0.024).

**FIGURE 3:**
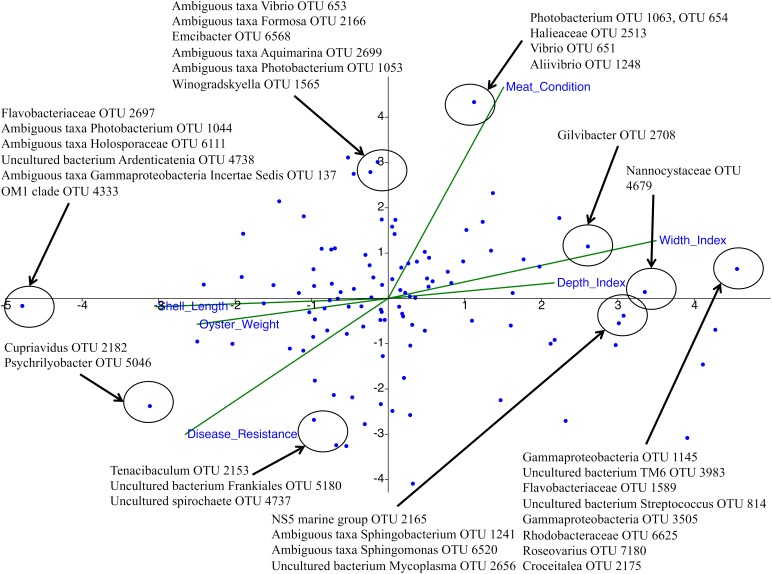
Canonical correspondence analysis (CCA) plot using 3% relative abundance filtered data. *Cupriavidus*, *Psychrilyobacter*, *Tenacibaculum*, and *Frankiales* were found to be strongly associated with OsHV-1 μvar disease-resistance, while OTUs assigned to the *Photobacterium*, *Vibrio*, and *Aliivibrio* were negatively associated with OsHV-1 μvar disease-resistance. Axis 1 and 2 were able to significantly represent 53.2% of the data (*p* = 0.001 for both axes with 999 permutations).

A CCA was used to highlight associations between specific OTUs, OsHV-1 μvar disease-resistance and EBVs of other traits (Figure 3). OTUs matching the *Cupriavidus* (OTU 2182) and *Psychrilyobacter* (OTU 5046) genera were closely coupled with disease-resistance, followed by a member of the *Tenacibaculum* (OTU 2153) genus and an uncultured bacterium in the *Frankiales* order (OTU 5180). While OTUs assigned to members of the *Photobacterium* (OTU 1063; OTU 654; OTU 1053), *Vibrio* (OTU 651; OTU 653) and *Aliivibrio* (OTU 1248) genera were negatively associated with disease-resistance, but strongly associated with meat condition. Furthermore, members of the *Streptococcus* (OTU 814) and *Roseovarius* (OTU 7180) genera were closely associated with depth and width index, and also negatively associated with disease-resistance. The community composition was largely influenced by the first axis, driven by growth related EBVs. A MINE analysis identified a negative correlation between disease resistance and width index (*p* = 0.047; linear regression = -0.34), and a positive correlation between disease resistance and oyster weight (*p* = 0.038; linear regression = 0.15). Shell length and depth index had the strongest negative correlation (*p* = < 0.001; linear regression = -0.92), while oyster weight and shell length had the strongest positive correlation (*p* = 0.002; linear regression = 0.74; Supplementary Table 5).

### Defining the Core *C. gigas* Microbiome Across Different Resistance Lines

Due to the dynamic nature of oyster microbiomes, identifying a core microbiome can provide insights into which members may be driving the within-microbiome interactions and possibly shaping the community composition. While we were unable to identify a universal core microbiome across all samples, analyses of individual families revealed that each family had a small core microbiome (9–109 OTUs), with many of these OTUs shared across families. Families 30 and 84, within RG2, shared the most core OTUs (4) (Supplementary Figure 3). In contrast, family 19 of RG2 had the most unique core OTUs (27), that is those core OTUs not shared with any other family. To determine how many unique core OTUs were present in each oyster family (and therefore each RG), we compiled all of the core OTUs from the core analysis and removed duplicate bacteria. When doing this, a total of 9, 54, and 16 unique OTUs were assigned to RG1, RG2, and RG3 respectively (Table 2). When performing a separate core analysis on each RG as a whole, RG1 was comprised of two core members, a member of the *Winogradskyella* genus (OTU 1511) and a member of the *Bradyrhizobiaceae* family (OTU 6417). While, no core bacterial members were found for RG2 or RG3 microbiomes.

**Table 2:** Unique core bacterial members from individual oyster families collated into their respective resistance groups (RG).

Resistance group	Combined unique core members from individual family lines	
RG1	Acinetobacter OTU_2667 Ambiguous taxa Cellulophaga OTU_3456 Brevundimonas OTU_6676 Ambiguous taxa Marinomonas OTU_1295 Ambiguous taxa Ilumatobacter OTU_4817	Rhodobacteraceae OTU_6650 Ambiguous taxa Marinomonas OTU_1295 Roseobacter OTU_6715 Rhodobacteraceae OTU_7212
RG2	Uncultured Salinimonas OTU_6618 Planctomycetaceae OTU_4123 Ambiguous taxa Gammaproteobacteria Incertae Sedis OTU_465 Uncultured bacterium Anaerolineaceae OTU_4681 Uncultured bacteria Gammaproteobacteria OTU_5902 Croceitalea OTU_2175 Uncultured bacterium Halanaerobiales ODP1230B8.23 OTU_4816 Oceanospirillaceae OTU_1577 Pseudoalteromonadaceae OTU_3257 Uncultured bacterium Ilumatobacter OTU_4687 Uncultured bacterium Acidobacteria Subgroup 21 OTU_5711 Rhodobacteraceae OTU_6481 Ambiguous taxa Acidobacteria Subgroup 9 OTU_7 Persicirhabdus OTU_866 Uncultured bacterium Ralstonia OTU_1255 Flavobacteriaceae OTU_1551 Ambiguous taxa Profundimonas OTU_1559 Ambiguous taxa JTB255 marine benthic group OTU_1588 JTB255 marine benthic group OTU_1642 Gilvibacter OTU_2167 Myxococcales OTU_238 Ambiguous taxa Flavobacteriaceae OTU_3100 Uncultured bacterium Pir4 lineage OTU_3294 Halieaceae OTU_3519 Uncultured bacterium OM1 clade OTU_4096 Planctomycetaceae OTU_4100 Ambiguous taxa OM1 clade OTU_4271 Planctomycetaceae OTU_4278	Sva0725 OTU_433 Uncultured bacterium OM1 clade OTU_4332 Uncultured bacterium Sva0996 marine group OTU_4477 Ambiguous taxa Ilumatobacter OTU_4840 Ambiguous taxa Sva0996 marine group OTU_4982 Uncultured bacterium Ardenticatenia OTU_5150 Rhodobiaceae OTU_6148 Rhodobacteraceae OTU_6485 Sphingomonadales OTU_6620 Marivita OTU_6626 Beijerinckiaceae OTU_6687 PAUC43f marine benthic group OTU_699 Anderseniella OTU_7187 Ambiguous taxa Sandaracinaceae OTU_1154 Ambiguous taxa Thiogranum OTU_1467 Ambiguous taxa Holophagae Subgroup 23 OTU_251 Candidatus Thiobios OTU_6098 JTB255 marine benthic group OTU_1566 OM190 OTU_2018 Uncultured bacterium Holophagae Subgroup 23 OTU_240 Uncultured bacterium Belgica2005-10-ZG-3 OTU_25 Ambiguous taxa Roseibacillus OTU_2529 OM60 (NOR5) clade OTU_3504 Uncultured bacterium Desulfobulbus OTU_404 Ambiguous taxa Sva0996 marine group OTU_4976 Ambiguous taxa Acidobacteria Subgroup 17 OTU_785
RG3	Rhizobiales OTU_6486 Uncultured bacterium Rickettsiaceae OTU_5903 Vibrionaceae OTU_655 Ambiguous taxa Sphingobacterium OTU_1241 Ambiguous taxa NS4 marine group OTU_2698 Pseudomonas OTU_3032 Gammaproteobacteria OTU_3505 Roseovarius OTU_7180 Uncultured bacterium Maribacter OTU_1486	Desulfobulbus OTU_235 Uncultured bacterium Emcibacter OTU_6286 Rhodobacteraceae OTU_7097 Mycoplasma OTU_3722 Uncultured bacterium Phyllobacteriaceae OTU_6619 Ruegeria OTU_6653 Uncultured bacterium Rhodobacteraceae OTU_7173


*Each family was found to have a core microbial community, the displayed core OTUs are those not shared with any other family line. RG1 is the most disease RG, RG2 is an intermediate RG, and RG3 is the most disease susceptible group*.

## Discussion

The principal goal of this study was to identify patterns in the *C. gigas* microbiome across 35 oyster families with differing levels of resistance to OsHV-1 μvar disease, with the objective of elucidating microbial taxa associated with disease resistance. Immunosuppression from OsHV-1 μvar infection allows opportunistic bacteria within the oyster’s microbiome to induce bacteremia, killing the host (Petton et al., 2015; de Lorgeril et al., 2018). Characterizing these interactions and gaining insights into the oyster microbiome is essential to further understand the dynamic interplay between the microbiome, OsHV-1 μvar and disease. A significant difference in the structure of the microbiome of oysters exhibiting different levels of resistance to OsHV-1 μvar disease was observed. Specifically, the microbiomes associated with the oysters showing the most resistance to OsHV-1 μvar disease (RG1) and moderately resistant oysters (RG2) were significantly different to the most disease susceptible (or least resistant) group (RG3). When considering disease resistance, we observed a strong negative association between the OsHV-1 μvar disease resistance of oyster hosts and the occurrence of OTUs assigned to the *Vibrio* (OTUs 651 and 653), *Photobacterium* (OTUs 1063, 654, and 1053), *Aliivibrio* (OTU 1248), *Streptococcus* (OTU 814) and *Roseovarius* (OTU 7180) genera, while on the other hand, the microbiomes of the most resistant families had an over-representation of OTUs assigned to the *Cupriavidus* (OTU 2182), *Psychrilyobacter* (OTU 5046), and *Tenacibaculum* (OTU 2153) genera.

The association between the occurrence of *Vibrio* and disease susceptibility was further supported by a significant elevation of an uncharacterized member of the *Vibrio* in RG3, and the results of a *Vibrio*-specific qPCR assay. These results are consistent with growing evidence implicating a role of the *Vibrio* community in oyster disease (Sugumar et al., 1998; Waechter et al., 2002; Garnier et al., 2007; Saulnier et al., 2010; Lemire et al., 2015; Petton et al., 2015; Green et al., 2018; King et al., 2018). Specifically, there is previous evidence that prior to oyster disease onset, the native *Vibrio* community is replaced by pathogenic *Vibrio* species (Lemire et al., 2015). Further, in corals, small shifts in the *Vibrio* community are sufficient to shift the microbiome metabolism (Thurber et al., 2009). Our data provides a new perspective on this interaction, whereby the total load of *Vibrio* differed between disease susceptible and resistant oyster families. This is supported by a recent study, which demonstrated that the *Vibrio* load following OsHV-1 μvar infection was significantly higher in disease-susceptible oysters (de Lorgeril et al., 2018). An increased *Vibrio* community size may provide further potential for pathogenic species to replace benign colonizers. On the other hand, a higher background load of *Vibrio* may become important under periods of stress, such as with OsHV-1 μvar infection, resulting in duel infection, as has recently been described (de Lorgeril et al., 2018). This is also indirectly supported by a previous study which observed reduced mortality in OsHV-1 infected oysters that were treated with antibiotics (Petton et al., 2015).

Increases in the abundance of OTUs assigned to the *Photobacterium* genus, as were observed here, often co-occur with an increase in the *Vibrio* community in oyster microbiomes (Wegner et al., 2013; Lokmer and Wegner, 2015). While members assigned to this genus have been identified as pathogens of other aquatic organisms (Pedersen et al., 2009; Liu et al., 2016), to our knowledge, no species of *Photobacterium* has been identified as an oyster pathogen. Members of the *Streptococcus* and *Aliivibrio* genera are known pathogens of fish and crabs (Pappalardo and Boemare, 1982; Egidius et al., 1986; Creeper and Buller, 2006; Urbanczyk et al., 2007), while a member of the *Roseovarius* genus is the causative agent of roseovarius oyster disease (formally juvenile oyster disease) in *Crassostrea virginica* (Boettcher et al., 2005; Maloy et al., 2007), yet to our knowledge these genera have not been implicated in disease of *C. gigas* previously, despite being over-represented in the most disease susceptible oyster families.

On the other hand, a strong positive association was observed between levels of disease resistance and the occurrence of OTUs assigned to the *Cupriavidus* (OTU 2182), *Psychrilyobacter* (OTU 5046) and *Tenacibaculum* (OTU 2153). Currently, little is known about the role of these genera in oysters. *Cupriavidus* species are commonly isolated from plants and soil (Cuadrado et al., 2010; Estrada-De Los Santos et al., 2014), but members of the *Psychrilyobacter* and *Tenacibaculum* have previously been observed in *C. gigas* microbiomes (Lee et al., 2009; Fernandez-Piquer et al., 2012; Wegner et al., 2013). *Psychrilyobacter* was observed in *C. gigas* microbiomes from Tasmania, Australia (Fernandez-Piquer et al., 2012), which is perhaps notable given that the oysters used in this study were initially sourced from Tasmania. In addition, we have previously identified an over-representation of a *Tenacibaculum* OTU in oyster microbiomes that were unaffected by a summer mortality outbreak (King et al., 2018).

As already stated, a significant elevation of OTUs belonging to the *Vibrio* and *Photobacterium* genera abundance in disease susceptible oysters has also been previously observed (de Lorgeril et al., 2018), supporting our findings. However, while we identified members of the *Psychrilyobacter* and *Tenacibaculum* genera to be associated with disease resistance, the same study (de Lorgeril et al., 2018) observed an increase in these same genera in an experimental infection experiment using disease susceptible oysters. Differences in bacterial taxa abundance and taxonomic assignment could be attributed to contrasting sequencing techniques and data analysis. For example, we used the V1–V3 hypervariable region, and clustered OTUs at the 97% identity level, compared to V3–V4 and having OTUs clustered at a three-nucleotide difference threshold (de Lorgeril et al., 2018). Furthermore, this study deployed oysters to the field, while the aforementioned study carried out their experiments in tanks. Tank based studies are known to significantly alter the oyster microbiome composition compared to oysters sourced from the environment (Lokmer et al., 2016a).

The oyster microbiome is dynamic in nature, changing in response to stressors such as disease, antibiotics, translocation, and heat (Wegner et al., 2013; Lokmer and Wegner, 2015; Lokmer et al., 2016b; de Lorgeril et al., 2018; Green et al., 2018; King et al., 2018). The microbiome assemblage can also be influenced by the oyster life stage, the genetics of the host oyster, and spatial location (Trabal et al., 2012; Wegner et al., 2013; Lokmer et al., 2016a; King et al., 2018). Because we only have one sampling point, our study would not capture the dynamic nature of the oyster microbiome, and thus the oyster microbiome could change before the onset of disease. To fully capture the importance of the taxa identified in this study, a temporal study in the field encompassing a disease outbreak would be needed. However, as disease outbreaks are often very sudden, capturing a disease outbreak in the environment can be difficult.

In addition to identifying OTUs that are over- or under-represented within the microbiomes of oysters with different levels of disease-resistance, another way to identify putatively important bacteria within the microbiome of a host organism involves the identification of “core” microbiome members (Ainsworth et al., 2015). Identifying which bacterial members are consistent and stable across microbial communities is important in unraveling the functional contribution of these core bacteria (Ainsworth et al., 2015). Notably, we could not define a universal core microbiome across all of the studied oyster families at the OTU level, suggesting significant heterogeneity in oyster microbiome structure, or possible differences in micro-geographic variation. However, we identified core microbiome members within each family microbiome, whereby a number of ‘unique’ core members often occurred exclusively in the core microbiome of a family. This is in accordance with previous observations that the composition of an oyster’s microbiome is partially governed by oyster genetics, particularly for shaping the rare specialist bacterial community (<1% abundance) (Wegner et al., 2013), although we have no information pertaining to the genetic differentiation between the studied oyster families. However, when examining the core microbiome across all of the families comprising the most highly disease-resistant group (RG1), we identified two core members, which included OTUs classified as members of the *Winogradskyella* genus (OTU 1511) and *Bradyrhizobiaceae* family (OTU 6417). OTUs assigned to the *Bradyrhizobiaceae* family have previously been observed in oysters (Sakowski, 2015), however, due to the coarse taxonomic assignment of this OTU, it is unclear what potential role this member of the *Bradyrhizobiaceae* family might have. *Winogradskyella* species are commonly found in numerous marine organisms, including oysters (Valdenegro-Vega et al., 2013; Park et al., 2015; Lee et al., 2017; Schellenberg et al., 2017; Franco et al., 2018), and are known for their role in amoebic-induced fish gill diseases (Embar-Gopinath et al., 2005, 2006). However, it is uncertain what function(s) *Winogradskyella* species play in oysters. We currently know little about the potential role, if any, of these core microbiome members in resistance, but these observations provide candidate target organisms for focused examinations of potential beneficial microbes within OsHV-1 μvar disease-resistance.

## Conclusion

We have shown that the microbiome of *C. gigas* displays significantly different microbial assemblage structure according to oyster disease-resistance. This study provides insights into the *C. gigas* microbiome within the context of oysters bred for disease-resistance and highlights the potential involvement of the oyster microbiome in disease-resistance. Members of the *Vibrio*, *Photobacterium*, *Aliivibrio*, *Streptococcus*, and *Roseovarius* genera were over-represented features of the microbiome of oysters with high OsHV-1 μvar disease susceptibility, which is consistent with previous studies implicating *Vibrio* in oyster disease dynamics. Furthermore, a significant elevation of *Vibrio* 16S rRNA gene copies in disease-susceptible oyster families could indicate a lack of immune response against *Vibrio* pathogens. However, further research is required to elucidate the role of these bacteria in oyster disease dynamics. Examination of ‘core’ bacteria identified species assigned to the *Winogradskyella* genus and *Bradyrhizobiaceae* family as core members of microbiomes assigned to RG1 and may also play a role in OsHV-1 μvar disease resistance. These results deliver evidence that the *C. gigas* microbiome differs between oysters with different levels of susceptibility to OsHV-1 μvar disease and identifies putative microbial determinants in disease onset and resistance.

## Data Availability

The datasets generated for this study can be found in NCBI SRA, PRJNA497763.

## Author Contributions

WK and KN carried out the fieldwork. WK and NW processed the samples. WK and NS analyzed the data. CJ, MD, WO’C, JS, and ML conceived and designed the study. TK produced the core microbiome analysis. WK, JS, and ML wrote the manuscript.

## Conflict of Interest Statement

The authors declare that the research was conducted in the absence of any commercial or financial relationships that could be construed as a potential conflict of interest.
