# Supplementary material for: Variability in the Composition of Pacific Oyster Microbiomes Across Oyster Families Exhibiting Different Levels of Susceptibility to OsHV-1 μvar Disease
Source: Front Microbiol. 2019 Mar 11;10:473. doi: 10.3389/fmicb.2019.00473 (PMC6421512; doi:10.3389/fmicb.2019.00473)
Supplement: Supplementary file 1 [file Data_Sheet_1.docx]

Variability in the composition of Pacific Oyster microbiomes across oyster families exhibiting different levels of susceptibility to OsHV-1 μvar disease

William L King^1, 2^, Nachshon Siboni^2^, Nathan LR Williams^1^, Tim Kahlke^2^, Khue Viet Nguyen^1, 2^, Cheryl Jenkins^3^, Michael Dove^4^, Wayne O’Connor^4^, Justin R Seymour^2^, Maurizio Labbate^1*^

^1^University of Technology Sydney, The School of Life Sciences, Ultimo NSW 2007, Australia

^2^University of Technology Sydney, Climate Change Cluster, Ultimo NSW 2007, Australia

^3^NSW Department of Primary Industries, Elizabeth Macarthur Agricultural Institute, Menangle, New South Wales, Australia

^4^NSW Department of Primary Industries, Port Stephens Fisheries Institute, Port Stephens, New South Wales, Australia

*Corresponding author:

Postal address: University of Technology Sydney, PO Box 123, Broadway NSW, 2007, Australia

Telephone number: +61 2 9514 4064 14

Email: maurizio.labbate@uts.edu.au

**Supplementary Table 1** Information pertaining to the data analysis, including the number of reads before and after cleaning, and rarefaction at 6000 reads per sample.

| Information | Read/Observation number |
| --- | --- |
| Reads after trimming | 4847965 |
| Reads after cleaning | 3961808 |
| Observations (OTUs) after cleaning | 4188 |
| Samples before rarefaction | 175 |
| Reads after rarefaction | 1002000 |
| Observations (OTUs) after rarefaction | 4188 |
| Reads after filtering below 0.1% prevalence | 984544 |
| Observations (OTUs) after 0.1% filtration | 3294 |
| Samples after rarefaction | 167 |

**Supplementary Table 2** Number of reads per sample before rarefication

| F29_05: 731.0  F72_05: 1880.0  F10_01: 2033.0  F67_05: 2061.0  F19_01: 4128.0  F19_03: 4587.0  F62_01: 4597.0  F11_02: 5063.0  F16_04: 6230.0  F23_05: 6568.0  F16_05: 6577.0  F67_04: 6993.0  F10_03: 7114.0  F65_04: 7295.0  F27_01: 7373.0  F29_01: 7538.0  F86_01: 7590.0  F30_03: 7702.0  F15_04: 7736.0  F10_02: 7774.0  F19_05: 8045.0  F62_02: 8424.0  F29_04: 8540.0  F37_02: 8847.0  F65_05: 9150.0  F65_01: 9311.0  F10_04: 9389.0  F37_03: 9452.0  F67_02: 9781.0 | F30_01: 10205.0  F86_05: 10215.0  F80_05: 10223.0  F43_03: 10302.0  F07_02: 10462.0  F15_05: 10583.0  F35_01: 10795.0  F37_04: 11090.0  F86_04: 11283.0  F43_05: 11385.0  F66_01: 11654.0  F86_03: 11756.0  F15_03: 12194.0  F80_04: 12287.0  F03_05: 12362.0  F68_04: 12491.0  F77_01: 12839.0  F26_03: 12946.0  F19_04: 13207.0  F23_02: 13661.0  F72_04: 14037.0  F07_05: 14103.0  F23_01: 14296.0  F67_03: 14395.0  F30_04: 14551.0  F80_02: 14566.0  F80_03: 15462.0  F51_05: 15730.0  F84_04: 15775.0 | F43_04: 15877.0  F51_03: 15919.0  F26_05: 15990.0  F35_02: 16359.0  F23_03: 16468.0  F11_05: 16469.0  F62_05: 16607.0  F37_01: 17028.0  F68_05: 17041.0  F02_03: 17135.0  F07_04: 17364.0  F02_05: 17365.0  F65_02: 17554.0  F69_04: 17596.0  F35_03: 17972.0  F15_02: 17985.0  F30_05: 18074.0  F68_01: 18220.0  F11_04: 18400.0  F84_05: 18593.0  F36_03: 18782.0  F65_03: 19453.0  F69_02: 19735.0  F84_02: 19880.0  F77_02: 19980.0  F69_05: 20120.0  F51_04: 20441.0  F67_01: 20468.0  F25_05: 20541.0 | F77_03: 20678.0  F36_05: 20746.0  F66_02: 20872.0  F11_03: 21110.0  F36_02: 21131.0  F69_01: 21272.0  F07_03: 21634.0  F51_02: 21682.0  F23_04: 21743.0  F66_05: 21827.0  F02_04: 21901.0  F25_02: 21910.0  F62_03: 22024.0  F25_04: 22134.0  F27_03: 22176.0  F84_03: 22216.0  F20_05: 22284.0  F77_04: 22291.0  F39_03: 22562.0  F10_05: 22582.0  F86_02: 22626.0  F62_04: 22643.0  F25_03: 22824.0  F80_01: 22928.0  F68_03: 23996.0  F66_03: 24114.0  F68_02: 24349.0  F19_02: 24435.0  F20_02: 24597.0 | F84_01: 24949.0  F15_01: 25025.0  F07_01: 25612.0  F20_01: 25728.0  F25_01: 25940.0  F29_02: 26149.0  F72_01: 26169.0  F27_05: 26187.0  F03_04: 26265.0  F43_01: 26583.0  F69_03: 26794.0  F16_01: 27004.0  F26_01: 27084.0  F37_05: 27097.0  F161_01: 28055.0  F16_03: 28125.0  F77_05: 28252.0  F26_02: 28352.0  F66_04: 28784.0  F39_01: 29096.0  F51_01: 29221.0  F72_03: 29503.0  F35_05: 30062.0  F72_02: 30859.0  F26_04: 30959.0  F11_01: 31146.0  F35_04: 32978.0  F39_02: 33203.0  F30_02: 35308.0 | F02_02: 36633.0  F40_03: 37209.0  F20_04: 37217.0  F39_04: 37323.0  F16_02: 37486.0  F40_05: 37565.0  F03_03: 37590.0  F161_03: 37681.0  F161_05: 37814.0  F20_03: 39270.0  F02_01: 39548.0  F161_02: 41347.0  F39_05: 41547.0  F36_01: 43010.0  F161_04: 43782.0  F01_02: 46130.0  F36_04: 46154.0  F03_01: 46315.0  F40_01: 46664.0  F43_02: 46703.0  F27_04: 47122.0  F01_03: 48356.0  F01_05: 51053.0  F29_03: 52418.0  F40_04: 55924.0  F03_02: 57403.0  F27_02: 58607.0  F40_02: 69726.0  F01_04: 77185.0  F01_01: 78470.0 |
| --- | --- | --- | --- | --- | --- |

**
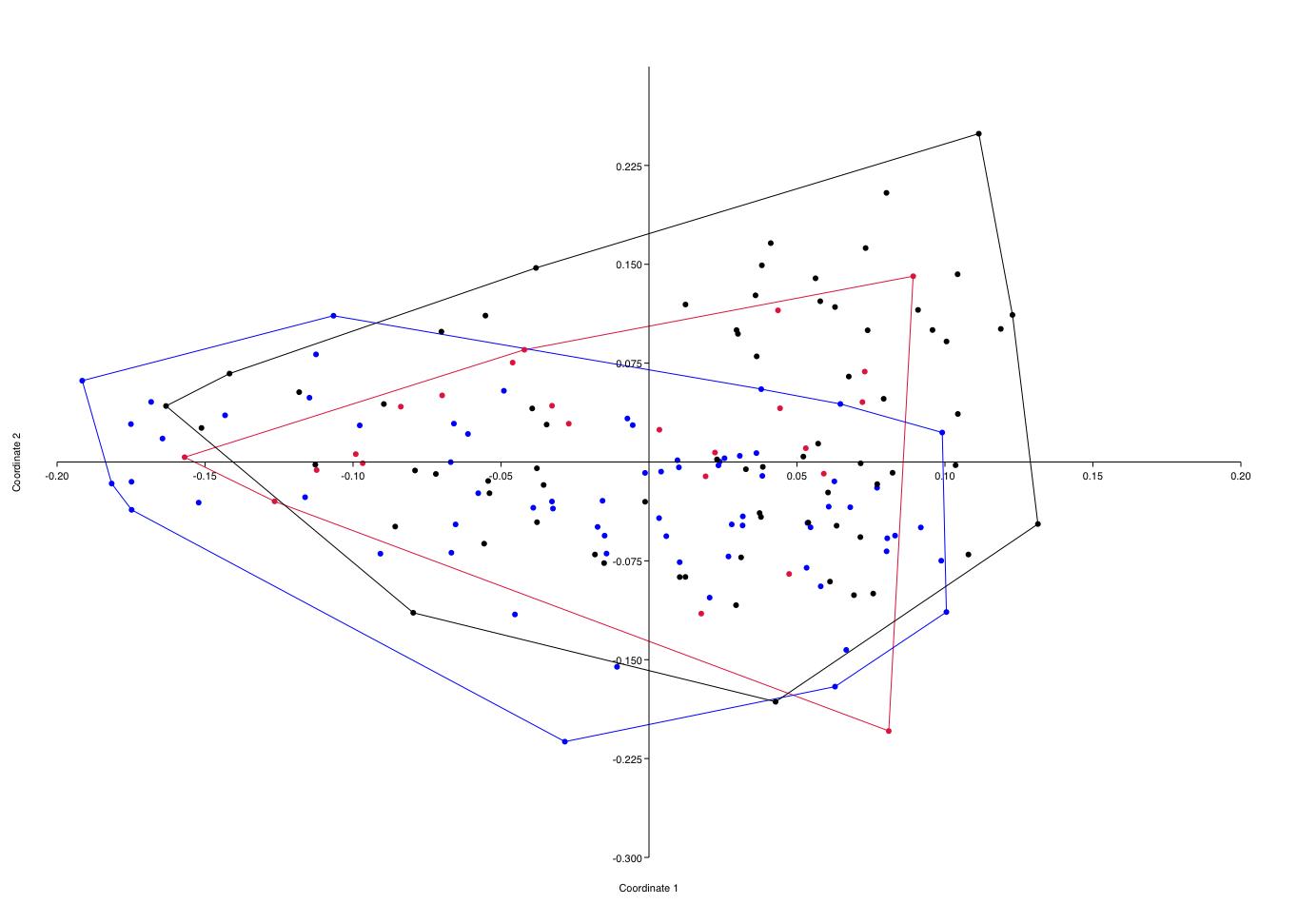
**

**Supplementary Figure 1** Principal Coordinates Analysis (PCoA) of oyster microbiomes with a Bray-Curtis similarity index. Blue dots are resistance group 1 (RG1) microbiomes, red dots are resistance group 2 (RG2) microbiomes, and black dots are resistance group 3 (RG3) microbiomes. Axes 1 and 2 represent 10.7% and 6.4% of the data respectively. Transformation exponent c = 6.

**Supplementary Table 3** SIMPER analysis of resistance group 1 (RG1) microbiomes compared to resistance group 3 (RG3) microbiomes. The top 10 OTUs are displayed with their dissimilarity contribution and transformed mean representation. Dissimilarity contribution is cumulative.

| OTU | Dissimilarity (%) | RG1 mean | RG3 mean |
| --- | --- | --- | --- |
| *Pseudomonas* 2034 | 2.204 | 1.02 | 2.05 |
| Uncultured bacterium *Psychrobacter* 1488 | 2.001 | 1.81 | 1.35 |
| Uncultured bacterium *Marispirillum* 6464 | 1.267 | 1.03 | 1.13 |
| Ambiguous taxa *Maribacter* 1117 | 1.16 | 1.28 | 1.29 |
| *Rhodospirillaceae* 6418 | 1.062 | 0.709 | 0.926 |
| *Winogradskyella* 1511 | 0.9522 | 1.42 | 1.1 |
| Uncultured bacterium *Mycoplasma* 3150 | 0.9466 | 0.598 | 0.891 |
| Uncultured bacterium *Rhodobacteraceae* 6466 | 0.9147 | 0.977 | 0.903 |
| Uncultured bacterium *Mycoplasmataceae* 680 | 0.8714 | 0.759 | 0.68 |
| Ambiguous taxa *Francisella* 2095 | 0.8521 | 0.766 | 1.14 |

**Supplementary Table 4** SIMPER analysis of resistance group 2 (RG2) microbiomes compared to resistance group 3 (RG3) microbiomes. The top 10 OTUs are displayed with their dissimilarity contribution and transformed mean representation. Dissimilarity contribution is cumulative.

| OTU | Dissimilarity (%) | RG2 mean | RG3 mean |
| --- | --- | --- | --- |
| *Pseudomonas* 2034 | 1.861 | 0.535 | 2.05 |
| Uncultured bacterium *Mycoplasma* 3150 | 1.547 | 1.63 | 0.891 |
| Uncultured bacterium *Psychrobacter* 1488 | 1.501 | 0.634 | 1.35 |
| Ambiguous taxa *Maribacter* 1117 | 1.263 | 1.37 | 1.29 |
| Uncultured bacterium *Rhodobacteraceae* 6466 | 1.196 | 0.965 | 1.13 |
| *Rhodospirillaceae* 6418 | 1.083 | 0.721 | 0.926 |
| Uncultured bacterium *Mycoplasmataceae* 680 | 1.07 | 1.09 | 0.68 |
| Ambiguous taxa *Francisella* 2095 | 0.9425 | 0.991 | 1.14 |
| *Winogradskyella* 1511 | 0.8938 | 1.33 | 1.1 |
| Uncultured bacterium *Rhodobacteraceae* 6466 | 0.841 | 0.872 | 0.903 |

**Supplementary Figure 2** The total *Vibrio* load determined with a *Vibrio* specific 16S qPCR assay. Blue columns represent the average *Vibrio* load in a family, while red columns represent the average *Vibrio* load for the resistance group (RG). Error bars are standard error. Statistical comparisons between resistance groups are displayed on the resistance group mean values.

**Supplementary Table 5** Maximal information-based nonparametric exploration (MINE) analysis of expected breeding values (EBVs).

| X variable | Y variable | MIC | Linear regression | p-value |
| --- | --- | --- | --- | --- |
| Shell length | Depth index | 0.81363 | -0.9193912 | <0.001 |
| Oyster weight | Shell length | 0.59185 | 0.74213886 | 0.002 |
| Width index | Meat condition | 0.54716 | 0.44662884 | 0.006 |
| Width index | Depth index | 0.54643 | 0.7001985 | 0.006 |
| Width index | Shell length | 0.54168 | -0.8329716 | 0.007 |
| Oyster weight | Meat condition | 0.49895 | -0.3160977 | 0.02 |
| Disease resistance | Oyster weight | 0.45763 | 0.1452698 | 0.04 |
| Shell length | Meat condition | 0.45451 | -0.2803066 | 0.04 |
| Oyster weight | Width index | 0.45087 | -0.5962815 | 0.04 |
| Disease resistance | Width index | 0.44843 | -0.3397912 | 0.05 |

**Supplementary Figure 3** Upset figure showing core microbes in each oyster family. Numbers of OTUs are displayed in a column chart. Unconnected dots represent unique OTUs only seen in that family line. Connected dots represent OTUs that are present in more than one family line. UpSet figure was made using the UpSetR package (Conway et al., 2017) in the R statistical environment (R_Core_Team, 2017).

Conway, J.R., Lex, A., and Gehlenborg, N. (2017). UpSetR: An R Package for the Visualization of Intersecting Sets and their Properties. *Bioinformatics*. 10.1093/bioinformatics/btx364

R_Core_Team (2017). R: A language and environment for statistical computing. *R Foundation for Statistical Computing, Vienna, Austria*.
